# C-doped ZnO decorated with Au nanoparticles constructed from the metal–organic framework ZIF-8 for photodegradation of organic dyes

**DOI:** 10.1039/c8ra09985e

**Published:** 2019-04-26

**Authors:** Qiang-Qiang Chang, Yi-Wei Cui, Hai-Huan Zhang, Fei Chang, Bao-Hua Zhu, Shi-Yong Yu

**Affiliations:** Inner Mongolia Key Laboratory of Chemistry and Physics of Rare Earth Materials, School of Chemistry and Chemical Engineering, Inner Mongolia University Hohhot 010021 Inner Mongolia China syyunano@imu.edu.cn

## Abstract

Recently, engineering metal–organic frameworks (MOFs) into metal oxides by solid state thermal decomposition has attracted wide attention for photocatalytic applications. Here, a series of C-doped ZnO materials decorated with Au nanoparticles (Au/C-ZnO) were constructed *via* controlled pyrolysis of ZIF-8 adsorbing different amounts of HAuCl_4_·4H_2_O. In this pyrolysis process, ZIF-8 was transformed into C-doped ZnO according to the EDX and XPS analysis. Meanwhile, HAuCl_4_·4H_2_O was transformed into Au nanoparticles that were uniformly dispersed on the surface of C-ZnO as seen in TEM images. The photocatalytic activity of as-prepared catalysts was evaluated by the degradation of methyl orange under UV-vis light irradiation. It was found that the photocatalytic activity of Au/C-ZnO was better than C-ZnO and pure ZnO. Furthermore, Au/C-ZnO exhibited high photocatalytic stability. After three consecutive cycles, there was no noticeable deactivation in the reaction. This unusual photocatalytic activity was attributed to the synergistic effect of C-doping and Au NPs.

## Abstract 2

C-doped ZnO decorated with Au nanoparticles (Au/C-ZnO) were prepared *via* one step pyrolysis of ZIF-8 adsorbing HAuCl_4_.

## Graphical abstract

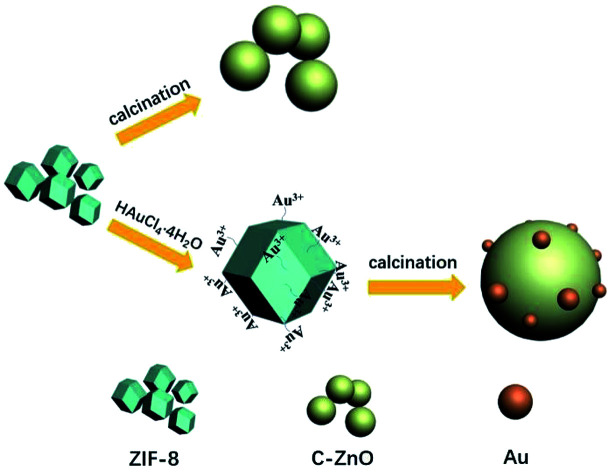

## 1. Introduction

ZnO has been extensively studied for the photocatalytic degradation of organic dyes due to its excellent optical, electrical, catalytic activity, chemical stability and environmental friendliness. However, ZnO has a wide band gap (∼3.3 eV) which can only be activated by ultraviolet (UV) light. Moreover, its rapid recombination rate of charge carriers limits its significant practical application in the photocatalysis field.^1^ Considerable efforts have been dedicated to improving the visible light response capacity and restrain recombination of photoinduced electron–hole pairs of single ZnO such as doping with metals and nonmetals, creating structural vacancies and combining with small-bandgap semiconductors.^2–4^

Among these, C-doped ZnO (C-ZnO) as an emerging visible-light-responsive photocatalyst has attracted wide attention because C doping can narrow the band gap of ZnO by creating an intermediate energy level just above the valence band of ZnO.^5^ Importantly, C doping can also promote the separation of charge pairs.^6^ C-doped ZnO can be prepared by situ synthesis or post-treatment. However, the situ doping requires a critical synthesis condition, while post treatment leads to C only distribution of the surface of ZnO.^7^

In addition, combining the plasmonic metal nanoparticles (NPs) with ZnO has been regarded as an effective approach to enhance the photocatalytic efficiency of the ZnO. On one hand, noble metal NPs have been shown to increase the efficiency of charge carrier separation of ZnO by forming Schottky junction with ZnO.^8^ On the other hand, the surface plasmon resonance (SPR) effect of noble metal NPs can induced light absorption and forming stronger electronic filed.^9^

Recently, metal–organic-frameworks (MOFs) as precursors derived metal oxides have drawn considerable interest. Because the metal oxides derived from MOFs were inherited the porous structures of MOFs.^10^ Moreover, carbon modified metal oxides can be synthesized by using MOFs as precursor.^11^ ZIF-8 as a Zn-containing MOF can be easily synthesized using a rapid room temperature route without a stabilizing agent or activation processes.^12^

**Fig. 1:**
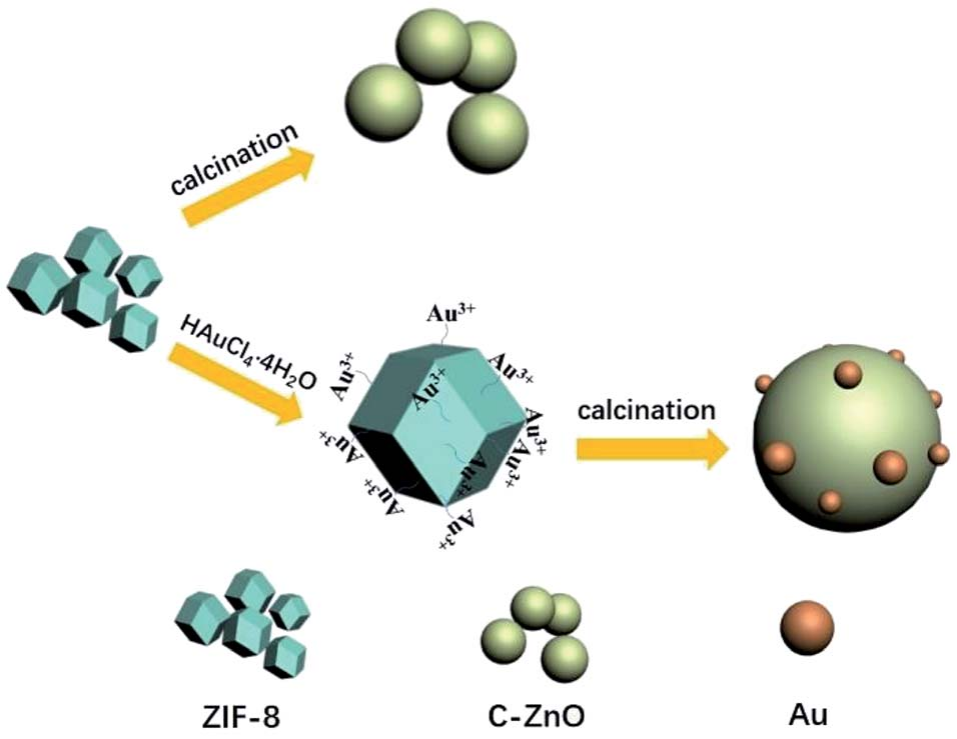
Schematic illustration of ZIF-8 derived C-ZnO, Au/C-ZnO.

In this paper, a series of Au nanoparticles decorated C-ZnO photocatalysts were constructed by simple one step solid-state pyrolysis of ZIF-8 adsorbing different amount of HAuCl_4_·4H_2_O mixture (Fig. 1). The characteristics of the obtained photocatalysts were analyzed and the photocatalytic activities of the samples were evaluated by the photocatalytic degradation of methyl orange under UV-vis light irradiation. Furthermore, our work proposed alternative way to synthesized C doping metal oxides and noble metal decorated metal oxides.

## 2. Experimental section

### 2.1 Chemicals

Zinc nitrate hexahydrate [Zn(NO_3_)_2_·6H_2_O ≥ 99.0%], absolute methanol, ethyl alcohol [EtOH≥99.7%] and chloroauric acid hydrated [HAuCl_4_·4H_2_O ≥ 98.0%] were purchased from Sinopharm Chemical Reagent Co., Ltd. Whereas 2-methylimidazole [H-MeIM] and methyl orange [MO ≥ 96%] were obtained from Aladdin. All chemicals were used directly without any further purification. Distilled water was used throughout the experiments.

### 2.2 Synthesis of ZIF-8 and its derived photocatalysts

ZIF-8 was synthesized according to a previously reported method.^13^ Firstly, Zn(NO_3_)_2_·6H_2_O (1 mmol) and H-MeIM (4 mmol) were dissolved in 25 mL methanol. Then the obtained solution was stirred 5 min and subsequently incubated without any interruption at room temperature for 24 h. C-ZnO photocatalyst was synthesized *via* direct calcination ZIF-8 at 450 °C for 3 h with the heating rate of 5 °C min^−1^. A series of Au nanoparticles decorated C-ZnO photocatalysts were prepared by following method: firstly, 0.5 g ZIF-8 powders were dispersed 20 ml ethanol followed by the addition of different volume HAuCl_4_·4H_2_O solution to make the ratio of Au and ZIF-8 as 0.2 wt%, 0.3 wt% and 0.4 wt%. The obtained suspension liquid was vigorous stirred for 60 min which make that the Au^3+^ sufficient adsorbed on the surface of ZIF-8. After that, the HAuCl_4_ modified ZIF-8 photocatalysts were collected by centrifugation and dried at 60 °C. Finally, the obtained samples were treated by the preparation process of C-ZnO. The as-prepared samples were denoted as Au/C-ZnO (0.2 wt%), Au/C-ZnO (0.3 wt%) and Au/C-ZnO (0.4 wt%) depending on the ratio of Au and ZIF-8. The reference sample of ZnO was obtained by directly calcining Zn(NO_3_)_2_·6H_2_O at 450 °C for 3 h.

### 2.3 Photocatalytic MO degradation

The photocatalytic activities of the samples were evaluated by the photocatalytic degradation of methyl orange using a xenon lamp (300 W) as simulated sunlight. The distance between the lamp and reactor was 10 cm. Typically, 50 mg catalyst was dispersed in a 100 ml 10 ppm MO solution. Prior to the degradation, the suspensions were continuously stirred in the dark for 30 min to achieve adsorption–desorption equilibrium of between catalysts and MO molecules. At every 10 min intervals, 3 ml suspensions was withdrawn, centrifuged and analyzed using UV-vis spectrometer at 462 nm.

### 2.4 Catalyst characterization

The phases of as prepared photocatalysts were characterized by X-ray power diffraction (XRD) on a Rigaku DMAX2500 X-ray diffractometer using a copper target. Particle sizes and morphologies of the samples were determined using transmission electron microscopy (TEM), high resolution transmission electron microscopy (HRTEM) on a JEM-2010 apparatus with an acceleration voltage of 200 kV and field-emission scanning electron microscopy (FE-SEM, HITACHI S-4800). Element distribution was obtained on energy-dispersive X-ray spectroscopy (EDX). UV-vis diffuse reflectance spectra (UV-DRS) of the samples were measured using a Lambda 750 UV/Vis spectrometer with BaSO_4_ act as the corrected baseline at room temperature. The photoluminescence (PL) spectra of the samples were recorded on Edinburgh Instruments FLS 920 spectrometer at an excitation wavelength of 320 nm. Surface composition and chemical states were analyzed with a Thermo Scientific Escalab 250Xi X-ray photoelectron spectroscope (XPS) equipped with Al Kα radiation, and the binding energy was calibrated by the C1s peak (284.6 eV) of the contamination carbon.

## 3. Results and discussion

### 3.1 Sample characterizations

**Fig. 2:**
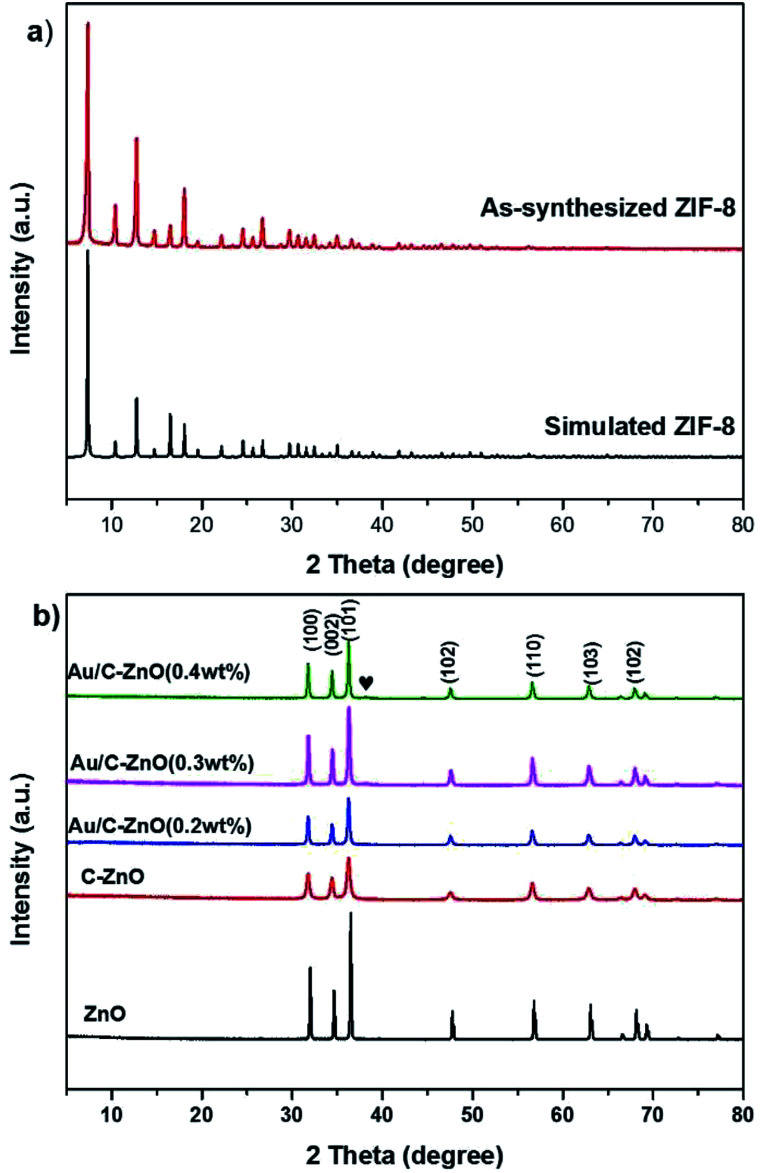
XRD patterns of simulated ZIF-8 and as-synthesized ZIF-8 (a); XRD patterns of ZnO, C-ZnO, Au/C-ZnO loaded different weight of Au (b).

The X-ray diffraction (XRD) patterns of ZIF-8, ZnO, C-ZnO and Au/C-ZnO are shown in Fig. 2. Fig. 2a shows that the XRD pattern of as-synthesized ZIF-8 is identified to be similar to simulated ZIF-8. In addition, the sharp and strong peaks of as-synthesized ZIF-8 indicate that it is well crystallized. Fig. 2b shows the XRD patterns of ZnO and ZIF-8 derived C-ZnO and Au/C-ZnO (*x*) (“*x*” symbol representing the mass ratio of Au and ZIF-8 of the precursor). After thermolysis, no ZIF-8 peaks could be found on the XRD patterns of ZIF-8 derived C-ZnO and Au/C-ZnO (*x*), suggesting that the crystalline phase of ZIF-8 has been completely vanished. The typical diffraction peaks of the all samples at 2*θ* 31.7, 34.4, 36.2, 47.5, 56.6, 62.8, 67.9° are ascribable to the (100), (002), (101), (102), (110), (103) and (102) reflections of ZnO (JCPDS 01-089-0511). In addition, a typical diffraction peak of Au NPs at 2*θ* 38.1° can be found in the XRD patterns of Au/C-ZnO (0.4 wt%). From the image of Fig. 2b, C-ZnO and Au/C-ZnO (*x*) shows weaker diffraction intensity than ZnO, implying that C-doped ZnO possesses lower crystallinity. Moreover, no diffraction peaks of carbon and other phase are observed in the XRD patterns of all samples.

**Fig. 3:**
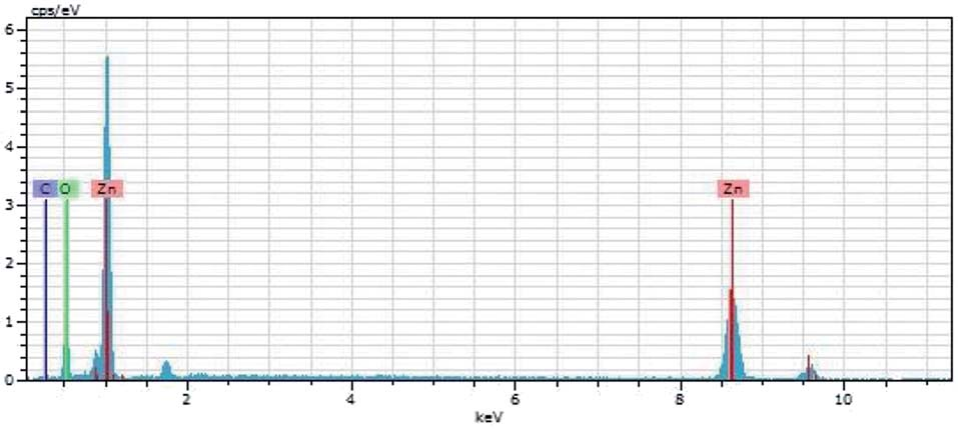
EDX pattern of the C-ZnO.

Fig. 3 shows the EDX pattern of the C-ZnO sample, conforming that the sample contains the obvious signals of Zn, O, and C elements. No diffraction peaks of carbon were found in the XRD patterns of C-ZnO which indicate that the carbon was doped in the lattice of ZnO.

**Fig. 4:**
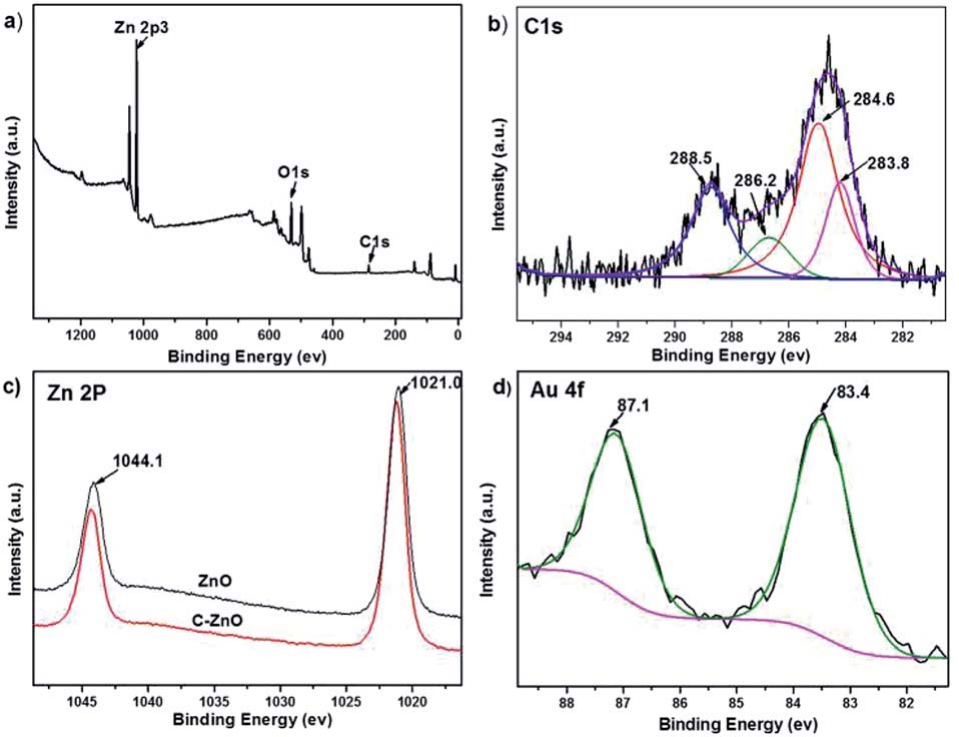
Survey (a) and C1s (b) XPS spectra of C-ZnO, Zn 2p XPS spectra of ZnO and C-ZnO (c) and Au 4f XPS spectra of Au/C-ZnO (0.3 wt%) (d).

XPS characterization were conducted to further verify the chemical composition and surface chemical state of the C-ZnO and Au/C-ZnO, as depicted in Fig. 4. The typical XPS survey spectrum of C-ZnO (Fig. 4a) shows the presence of the elements Zn, O and C. The carbon content is 4.34% (atomic%) depending on the XPS of C-ZnO. As shown Fig. 4b, the high-resolution spectra of C1s can be fitted four peaks at a BE of 283.8, 284.6, 286.2 and 288.5 eV. These peaks observed at different BE correspond to various types of carbon bonding in the sample. The main peak at 284.6 eV is attributed to the adventitious hydrocarbon. The peaks at 283.8 eV and 286.2 eV are due to Zn–O–C and Zn–C bonds, respectively.^14^ However, the peak locates at 288.5 eV is attributed to the adsorbed CO_2_ and structural carbonate species containing C=O. As shown Fig. 4c, BEs of 1021.0 eV and 1044.1 eV for bare ZnO were assigned to Zn 2p_3/2_ and 2p_5/2_. It can be clearly seen that the BE of Zn 2p in C-ZnO are blue shifted (0.2 eV) systematically relative to pure ZnO, which was due to the different chemical interactions present in both samples. The BE difference between Zn 2p_3/2_ and 2p_5/2_ XPS lines remains 23.1 eV for C-ZnO and ZnO, suggesting Zn is in Zn^2+^ state in the C-ZnO sample.^14^ The Au 4f XPS spectrum of Au/C-ZnO (0.3 wt%) was presented in Fig. 4d. Two peaks at the BEs of 83.4 and 87.1 eV, respectively, assigned to Au 4f_7/2_ and Au 4f_5/2_, indicating that the Au species in the sample were presented in the metallic state.^15^ From the EDX and XPS analyze, ZIF-8 was successfully transformed C doped ZnO during the pyrolysis process of ZIF-8.

**Fig. 5:**
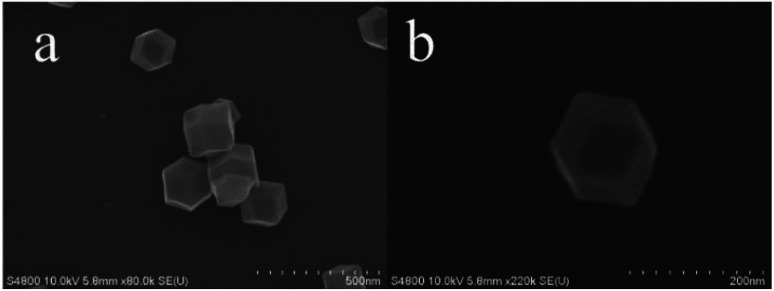
SEM images of ZIF-8.

**Fig. 6:**
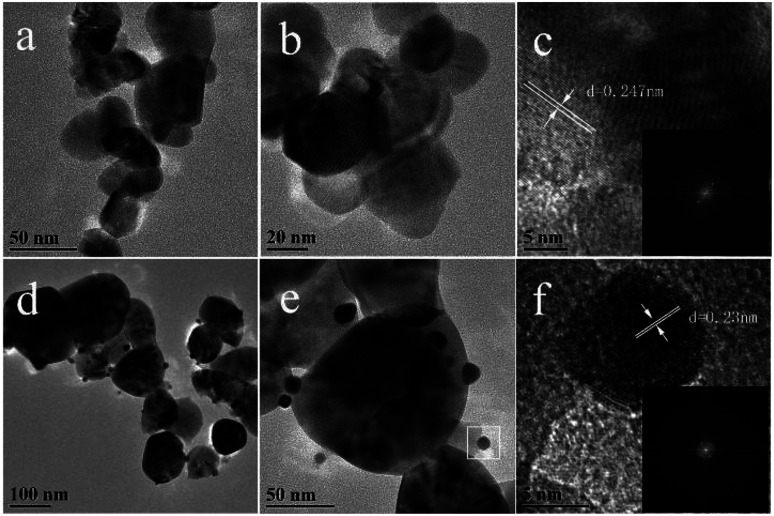
TEM (a and b) and HRTEM (c) images of C-ZnO (inset in the c is corresponding FFT image of c); TEM (d and e) and HRTEM (f) images of Au/C-ZnO (0.3 wt%) (inset in the f is corresponding to FFT image of f).

From the SEM of ZIF-8 (Fig. 5), it was exhibited as regular dodecahedral morphology with the size of ∼100 nm. TEM was used to further analyze the morphology and structures of the catalyst. Fig. 6a–f show the TEM images of C-ZnO and Au/C-ZnO (0.3 wt%). ZIF-8 derived C-ZnO are presented irregular nanoparticles with size of 50–150 nm (Fig. 6a and b). The HRTEM image of C-ZnO are presented in Fig. 6c in which the lattice spacing of *ca.* 0.247 nm is assigned to the (101) plane of ZnO. ZIF-8 derived Au/C-ZnO (0.3 wt%) is showed in Fig. 6d–f. The Au nanoparticles with the size of ∼10 nm can be found to be well dispersed on the surface of C-ZnO. The lattice spacing of *ca.* 0.23 nm is observed in Fig. 6f which corresponds to the (111) plane of Au, which indicates that the HAuCl_4_ molecules were decomposed into Au nanoparticles.

**Fig. 7:**
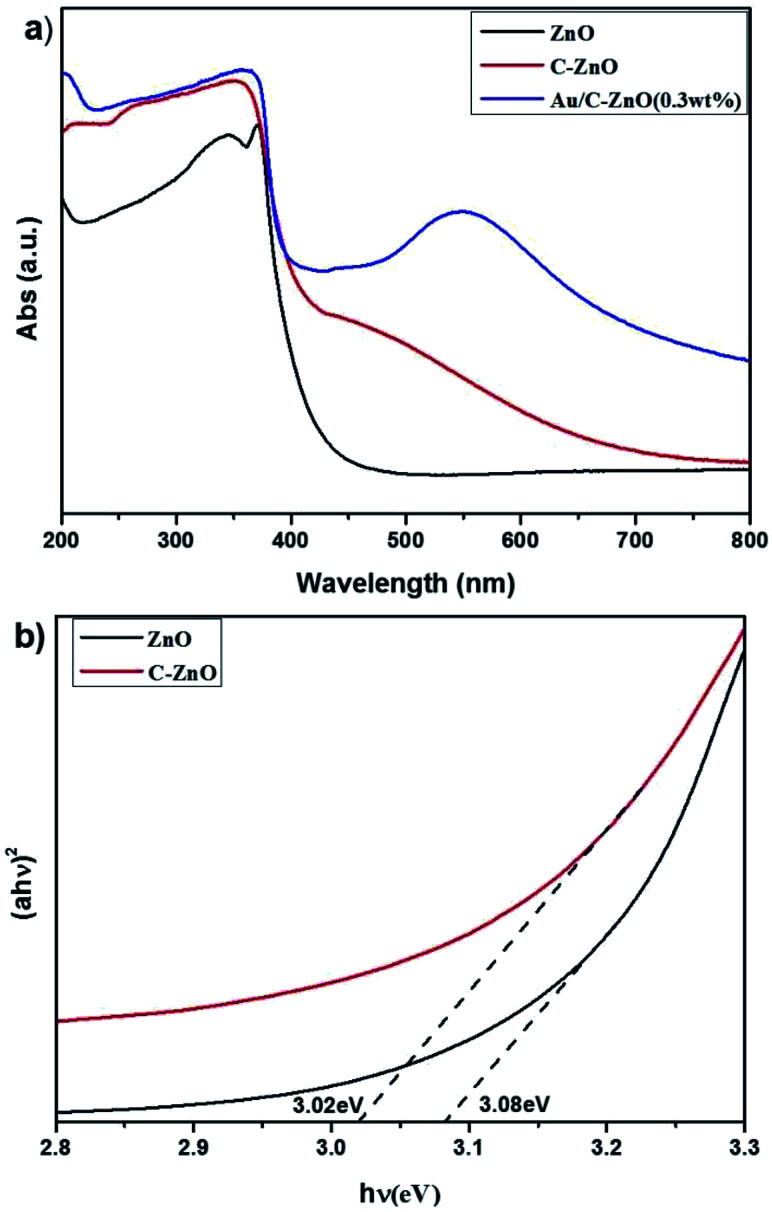
UV-vis diffuse adsorption spectra of the ZnO, C-ZnO and Au/C-ZnO (*x*) (a) and corresponding (*αhν*)^2^*vs. hν* plots for ZnO and C-ZnO (b).

UV-vis diffuse adsorption spectra (UV-DRS) of ZIF-8 derived C-ZnO and Au/C-ZnO (*x*) were shown in Fig. 7a. In comparison with ZnO, C-ZnO displays an obvious red-shift of optical bandgap absorption edge into visible-light region. The band gap energy (*E*_g_) of ZnO and C-ZnO was calculated from a plot of (*αhν*)^2^*vs.* (*hν*) according to Kubelka–Munk rule as shown in Fig. 7b. The band gap value of the ZnO and C-ZnO was measured about 3.08 eV and 3.02 eV, respectively. The narrowing band gap of C-doped ZnO is mainly induced by the C-doping. Z. Jiang *et al.*^16^ reported C doping not only expands the VB width of ZnO but also lifts its valance band maximum (VBM) energy. Furthermore, Au/C-ZnO (*x*) exhibited a higher absorption in the visible region compare to the C-ZnO which was due to the absorption of the Au surface plasmon resonance (SPR).

### 3.2 Photocatalytic degradation of MO

**Fig. 8:**
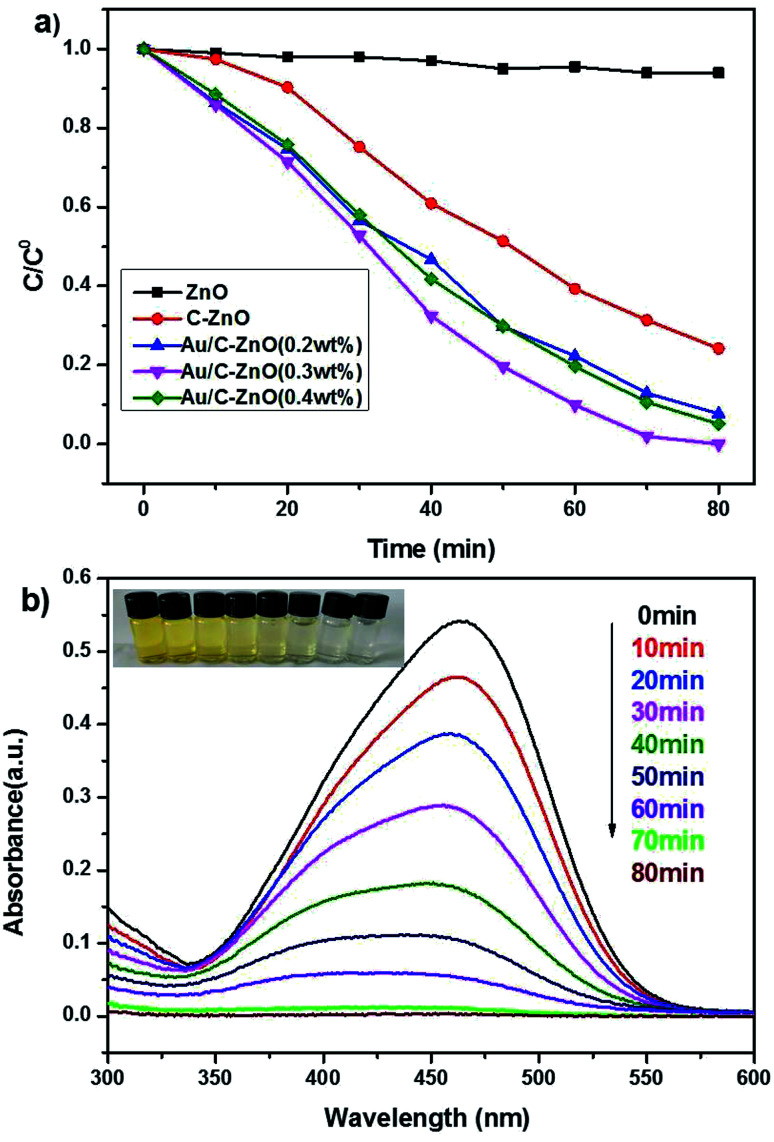
Photocatalytic degradation of MO by ZnO, C-ZnO and Au/C-ZnO (*x*) (a); time dependent UV-vis absorption spectrum showing the photocatalytic degradation of MO by Au/C-ZnO (0.3 wt%) (catalyst dose: 30 mg, 320 nm < *λ* < 780 nm, [MO]: 10 ppm, volume: 100 mL) (b).

Photocatalytic activity of the as-prepared catalysts was investigated by the photocatalytic degradation of MO using a xenon lamp (300 W) as simulated sunlight. As shown in Fig. 8a, the pure ZnO showed poor photocatalytic activity with only 6% MO degradation. However, C-ZnO showed 76% MO degradation in 80 min. Obviously, ZIF-8 derived Au/C-ZnO showed higher photocatalytic degradation of MO than C-ZnO and ZnO. It was also found that the photocatalytic activity of Au/C-ZnO was depended on the ratio of HAuCl_4_ and ZIF-8 of precursor. 92.3% and 95% of MO was degraded by the Au/C-ZnO (0.2 wt%) and Au/C-ZnO (0.4 wt%) in 80 min under light irradiation, respectively. However, 100% MO was degraded by the Au/C-ZnO (0.3 wt%) in only 70 min. This phenomenon also can be found in Fig. 8b in which the intensity of the absorption peak of MO is decreased gradually with the irradiation time increasing and the color of MO gradually changed from orange into no color.

### 3.3 Stability of photocatalyst

**Fig. 9:**
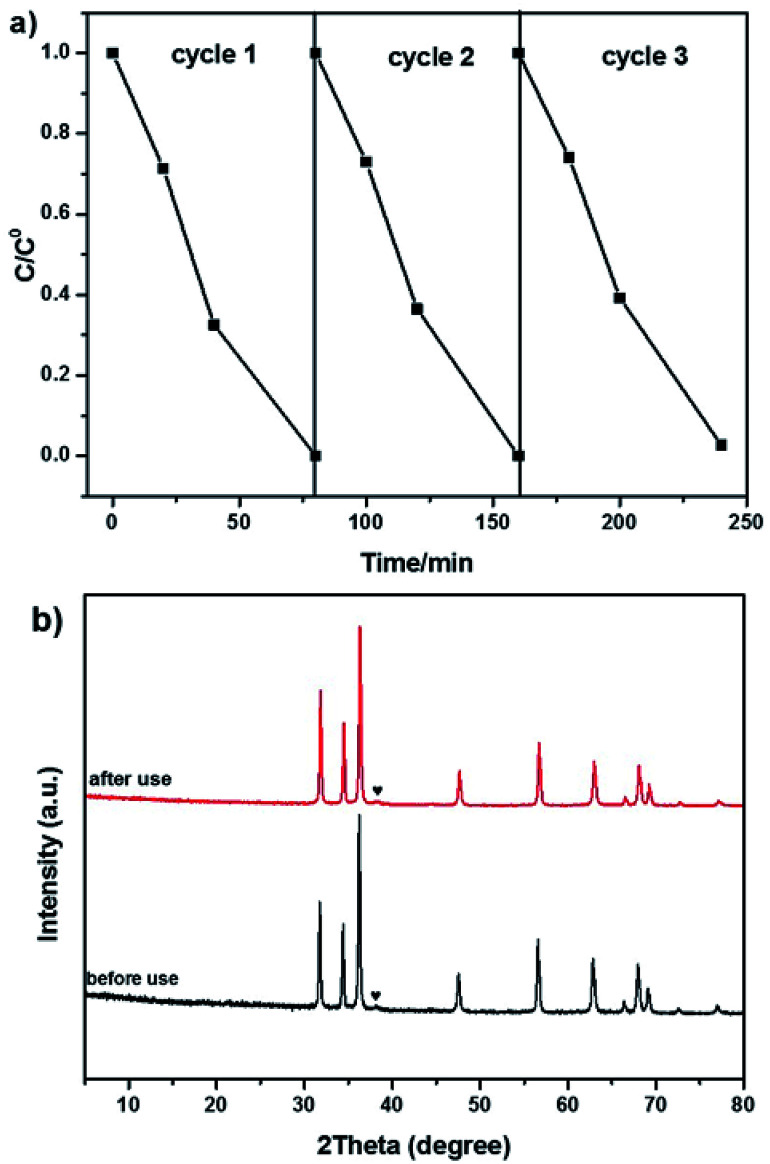
Cycling runs for the photocatalytic degradation of MO over Au/C-ZnO (0.3 wt%) (a); XRD patterns of Au/C-ZnO (0.3 wt%) before and after photocatalytic reaction (b).

To evaluate the stability of Au/C-ZnO (0.3 wt%), cycling experiments were carried out on MO degradation under light irradiation for three times. After three consecutive cycle's reactions, the photocatalytic activity of Au/C-ZnO (0.3 wt%) no significant decreased indicating the high stabilization of Au/C-ZnO (Fig. 9a). The outstanding stability of the ZIF-8 derived Au/C-ZnO was further determined by analyzing the XRD patterns of before and after reaction (Fig. 9b). The results revealed that phase structure of Au/C-ZnO (0.3 wt%) maintained intact after three recycling reaction.

### 3.4 Mechanism of enhanced photocatalytic activity

**Fig. 10:**
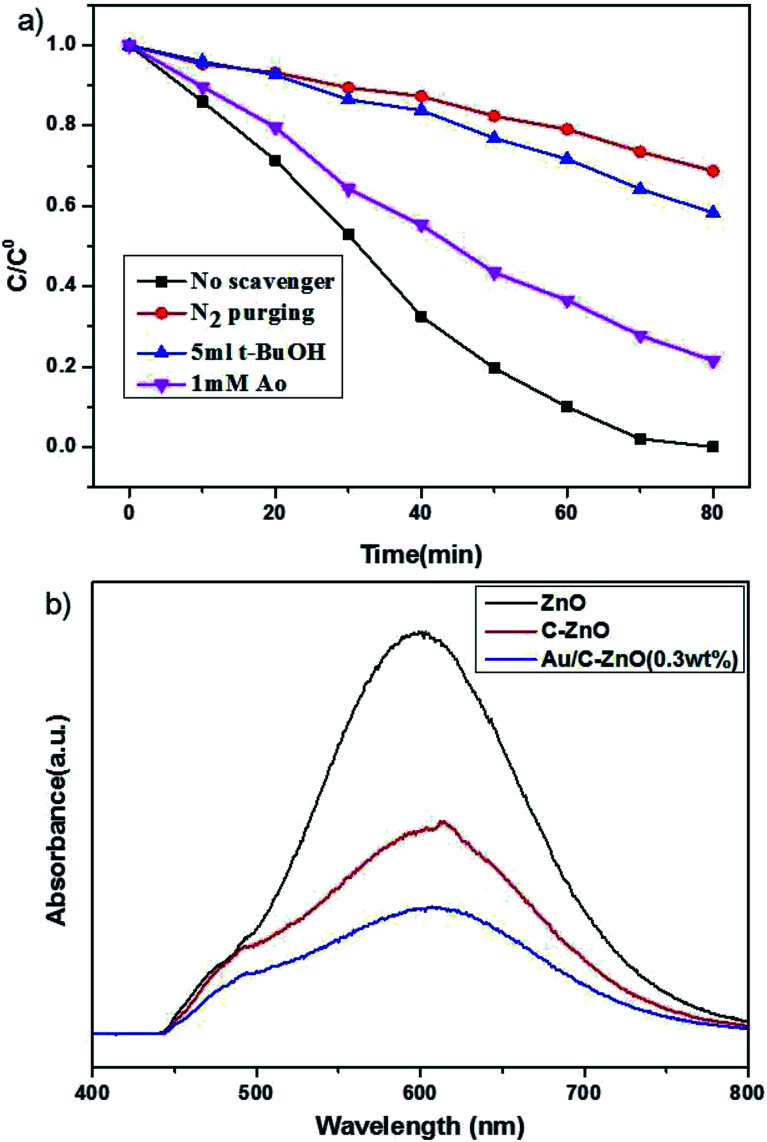
Influence of various scavengers on the photocatalytic activity of Au/C-ZnO (0.3 wt%) toward the degradation of MO (catalyst dose: 30 mg, 320 nm < *λ* < 780 nm, [MO]: 10 ppm, volume: 100 mL) (a); photoluminescence emission spectra of the ZnO, C-ZnO and Au/C-ZnO (0.3 wt%) (b).

In order to reveal the underlying reaction mechanism on the enhanced photocatalytic activity of Au/C-ZnO (0.3 wt%) in detail, the free radical and holes generation during the process of photocatalytic degradation of MO over Au/C-ZnO (0.3 wt%) were investigated by adding various scavengers or in the absence of O_2_. As shown in Fig. 10a, it can be seen that controlled experiment in the inert N_2_ atmosphere. A dramatic decreasing in the photocatalytic activity was observed indicating that the O_2_ or ·O_2_^−^ play an important role in the photocatalytic degradation process compared with in air atmosphere under light irradiation. When addition of *t*-BuOH as an ·OH scavenger^17^ and AO as an h^+^ scavenger,^18^ the photodegradation rate of MO also be suppressed suggesting that ·OH and h^+^ also participated the photodegradation process of MO.

The separation efficiency of charge carrier is one of the most key factors for photocatalytic activity. Photoluminescence (PL) spectra is always studied the photoelectron transfer and recombination.^19^ As shown in Fig. 10b, the main emission peak is observed at around 612 nm for the ZnO, C-ZnO and Au/C-ZnO (0.3 wt%) excited at 320 nm. The ZIF-8 derived C-ZnO exhibits a lower PL intensity than ZnO, because the C doping can accelerate the separation of photogenerated electron and hole pairs. In addition, ZIF-8 derived Au/C-ZnO (0.3 wt%) shows the lowest emission peak among the ZnO and C-ZnO, which indicated incorporation of Au NPs further lead to the charge transfer. The reduction of emission peak is due to the electron sink effect where the Au NPs act as electron trap to gather photoelectron of C-ZnO and suppress the recombination of photogenerated electron and hole pairs during illumination by forming a Schottky barrier between Au NPs and C-ZnO.

**Fig. 11:**
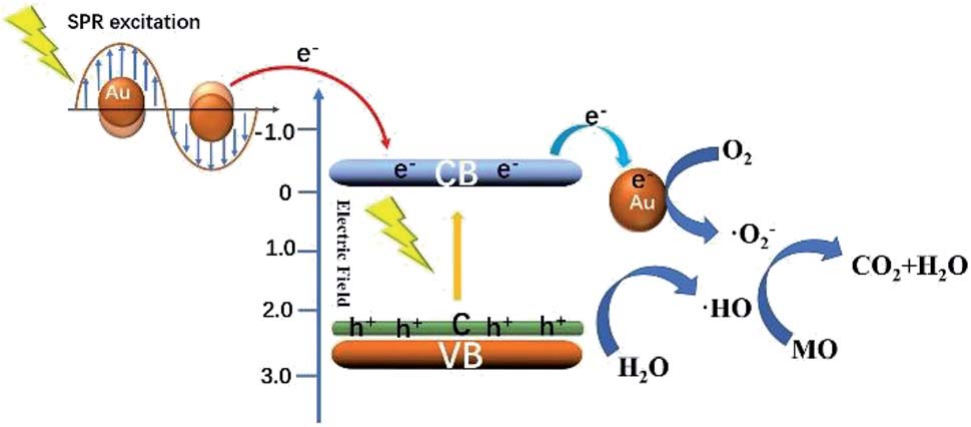
Schematic diagram showing the photocatalytic degradation MO over Au/C-ZnO under light irradiation.

Fig. 11 shows the schematic diagram for the photocatalytic degradation MO over Au/C-ZnO. When ZnO was doped C, the new additional bandgap electron states were appeared at VBM (valance band maximum) arising from C-doping, which expanded visible-light-response of ZnO.^16^ Under light irradiation, C-ZnO can be excited to promote the valence band electrons up to the conduction band and leaving holes in the valence band by part of visible light. Considering the lower Fermi level of Au NPs, the photo-generated electrons in the CB of C-ZnO can be easily transferred to Au NPs.^20^ In addition, the SPR of Au NPs can't be ignored for the significantly improved photocatalytic performance, which can be induced to generate energetic plasmonic electrons at around ∼550 nm visible light. Due to the high energy position of the SPR state of Au NPs, the excited energetic plasmonic electrons subsequently injected into the CB of C-ZnO.^21,22^ The photo-generated electrons and the excited energetic plasmonic electrons would react with adsorbed O_2_ to form ·O_2_^−^. However, the leaving holes in the valence band of C-ZnO would react with adsorbed OH^−^ or water molecule to form ·OH. The generated ·O_2_^−^, ·OH and h^+^ can mineralize MO molecules.

## 4. Conclusions

In summary, Au/C-ZnO hybrid nanostructures were fabricated by a-step calcination HAuCl_4_·4H_2_O treated ZIF-8. It was found that C was doped in ZnO lattice and Au NPs were uniformly loaded onto C-ZnO by this method. The as-prepared Au/C-ZnO showed enhanced photocatalytic degradation of MO than C-ZnO and ZnO. The improved photocatalytic activity was attribute to the synergistic effect of C-doping and Au NPs, where C-doping and SPR of Au NPs can improve the visible-light response of ZnO. Moreover, the formation of Schottky barrier between the C-ZnO and Au NPs can efficiently accelerate the electron transfer. The ratio of Au and ZIF-8 in the precursor also affected the photocatalytic activity. The optimal hybrid nanostructure for photocatalytic degradation of MO was determined to be Au/C-ZnO (0.3 wt%). Furthermore, Au/C-ZnO exhibited high photocatalytic stability. After three cycle's reactions, the Au/C-ZnO (0.3 wt%) showed no significant decrease on photocatalytic degradation of MO. The outstanding photocatalytic activity of Au/C-ZnO make it an effective way to degrade organic dyes.

## Conflicts of interest

There are no conflicts to declare.

## Supplementary Material

## References

[cit1] Pearton S. J., Norton D. P., Ip K., Heo Y. W., Steiner T. (2005). Superlattices Microstruct..

[cit2] Kubacka A., Fernández-García M., Colón G. (2012). Chem. Rev..

[cit3] Georgekutty R., Seery M. K., Pillai S. C. (2008). J. Phys. Chem. C.

[cit4] Chen D., Wang Z., Ren T., Ding H., Yao W., Zong R., Zhu Y. (2014). J. Phys. Chem. C.

[cit5] Lin Y. G., Hsu Y. K., Chen Y. C., Chen L. C., Chen S. Y., Chen K. H. (2012). Nanoscale.

[cit6] Yu W., Zhang J., Peng T. (2016). Appl. Catal., B.

[cit7] Liang P., Zhang C., Sun H., Liu S., Tade M. O., Wang S. (2017). Energy Fuels.

[cit8] C Damato T., Oliveira C. C. S. D., Ando R. A., Camargo P. H. C. (2013). Langmuir.

[cit9] Ha E., Lee L. Y. S., Man H. W., Tsang S. C. E., Wong K. Y. (2015). ACS Appl. Mater. Interfaces.

[cit10] Kaneti Y. V., Tang J., Salunkhe R. R., Jiang X., Yu A., Wu K. C. W., Yamauchi Y. (2017). Adv. Mater..

[cit11] Pan L., Muhammad T., Ma L., Huang Z. F., Wang S., Wang L. (2016). Appl. Catal., B.

[cit12] Cravillon J., Münzer S., Lohmeier S. J., Feldhoff A., Huber K., Wiebcke M. (2009). Chem. Mater..

[cit13] Venna S. R., Jasinski J. B., Carreon M. A. (2010). J. Am. Chem. Soc..

[cit14] Mishra D. K., Mohapatra J., Sharma M. K., Chattarjee R., Singh S. K., Varma S., Behera S. N., Nayak S. K., Entel P. (2013). J. Magn. Magn. Mater..

[cit15] Yang J., Wang X., Chen Y., Dai J., Sun S. (2015). RSC Adv..

[cit16] Alshammari A. S., Chi L., Chen X., Bagabas A., Kramer D., Alromaeh A., Jiang Z. (2015). RSC Adv..

[cit17] Jing L., Qu Y., Wang B., Li S., Jiang B., Yang L., Fu W., Fu H., Sun J. (2006). Sol. Energy Mater. Sol. Cells.

[cit18] Xu M., Niu H., Huang J., Song J., Mao C., Zhang S., Zhu C., Chen C. (2015). Appl. Surf. Sci..

[cit19] Singh G. P., Shrestha K. M., Nepal A., Klabunde K. J., Sorensen C. M. (2014). Nanotechnology.

[cit20] Hu J., You N., Yu Z., Zhou G., Xu X. (2016). J. Appl. Phys..

[cit21] He W., Kim H. K., Wamer W. G., Melka D., Callahan J. H., Yin J. J. (2014). J. Am. Chem. Soc..

[cit22] Lee J., Mubeen S., Ji X., Stucky G. D., Moskovits M. (2012). Nano Lett..

